# Esophageal Carcinoma Incidence and Survival Analysis: A Population-Based Cancer Registry Study in Central India

**DOI:** 10.7759/cureus.116626

**Published:** 2026-09-21

**Authors:** Pravinkumar V Ghongade, Bharat U Patil, Anupama Gupta, Swapna Maliye, Nitin Gangane

**Affiliations:** 1 Pathology, All India Institute of Medical Sciences, Nagpur, Nagpur, IND; 2 Pathology, Mahatma Gandhi Institute of Medical Sciences, Wardha, IND; 3 Pathology, All India Institute of Medical Sciences, Deoghar, Deoghar, IND

**Keywords:** esophageal cancer, hazard ratio, incidence rate, mortality rate, pbcr, prognosis, sensitivity analysis, survival outcome

## Abstract

Introduction: This study aimed to estimate the incidence, mortality, and overall survival (OS) of esophageal cancer (EC) and to evaluate the association of clinicopathological characteristics and treatment modality with OS.

Methods: A retrospective observational cohort study was conducted using data from the Population-Based Cancer Registry (PBCR) in rural central India. The dataset included 545 incident EC cases registered between 2010 and 2019, of which 476 were eligible for OS analysis. Incidence and mortality rates were estimated, OS was analyzed using the Kaplan-Meier method, and Cox proportional hazards regression was used to identify prognostic factors. Prespecified sensitivity analyses, including metastasis-adjusted and age-stratified Cox regression models, were performed to assess the robustness of the primary multivariable findings.

Results: The age-standardized incidence rate (ASIR) of overall EC cases was 3.99 per 100,000 population (95% CI: 3.50-4.47), while the age-standardized mortality rate (ASMR) was 3.29 per 100,000 population (95% CI: 2.94-3.64). The overall median survival was 178.33 days (95% CI: 115.49-234.48). The OS rates at 1, 3, 5, and 10 years were 31.3%, 19.7%, 17.4%, and 14.6%, respectively. OS differed significantly according to tumor site (χ² = 5.24, p = 0.043), treatment modality (χ² = 15.42, p = 0.031), and metastatic status (χ² = 162.64, p < 0.001) on Kaplan-Meier analysis. Median survival was 232 days (95% CI: 159.59-304.41) for localized disease, 199 days (95% CI: 166.38-231.61) for regional disease, 68 days (95% CI: 54.21-81.79) for distant disease, and 28 days (95% CI: 15.56-40.44) for advanced disease. On univariable Cox regression, patients with middle-third tumors had a lower hazard of death than those with upper-third tumors (HR: 0.74, 95% CI: 0.56-0.98; p = 0.036), and this remained significant after adjustment for metastatic status and in the age-stratified sensitivity model, while metastatic disease was associated with progressively higher mortality. Radiotherapy plus chemotherapy and surgery plus chemotherapy were independently associated with lower mortality after multivariable adjustment (HR: 0.48, 95% CI: 0.28-0.85, p = 0.011; HR: 0.45, 95% CI: 0.21-0.94, p = 0.035, respectively). Age, sex, and histological type were not significantly associated with OS.

Conclusion: EC demonstrated a substantial mortality burden and poor OS in this population-based cohort. Metastatic status was the strongest associated prognostic factor, while tumor site and treatment modality were associated with survival on Kaplan-Meier analysis. Radiotherapy plus chemotherapy and surgery plus chemotherapy remained independently associated with lower mortality after multivariable adjustment. These findings highlight the importance of early diagnosis, accurate assessment of disease extent, and appropriate multimodal management of EC.

## Introduction

Based on the 2022 Global Cancer Observatory (GLOBOCAN) estimates, esophageal cancer (EC) ranks as the 11th most frequently diagnosed malignancy globally and is the seventh leading cause of cancer mortality. In 2022, approximately 511,000 individuals were newly diagnosed with the disease, while an estimated 445,000 deaths were attributed to EC worldwide [1]. EC represents 2.6% of all newly diagnosed cancer cases worldwide, following colorectal (9.6%) and stomach (4.9%) cancers among the malignancies of the gastrointestinal tract [1].

EC is broadly classified into two principal histopathological subtypes: esophageal squamous cell carcinoma (ESCC) and esophageal adenocarcinoma (EAC) [2].

According to the GLOBOCAN 2022 estimates, EC represents a significant cancer burden in India, ranking seventh among all cancers in terms of incidence and sixth in cancer-related mortality. It accounts for approximately 3.9% of all newly diagnosed cancers and 5.4% of all cancer deaths in the country. In 2022, an estimated 58,686 new cases of EC were diagnosed in India, while approximately 56,258 deaths were attributed to the disease [3]. The incidence of EC is unevenly distributed across India, with particularly high rates reported from several states in the northeastern region [4].

Population-Based Cancer Registries (PBCRs) are sources that systematically record all newly diagnosed cancer cases in a defined population and provide high-quality epidemiological information for cancer surveillance. These registries generate data that are used to estimate incidence, prevalence, mortality, and survival of cancer, and they also allow for the evaluation of time trends and geographic variation in disease burden. PBCRs guide cancer prevention and control strategies, inform health-care policy, allow rational allocation of resources, and provide a valuable platform for assessing the long-term outcomes of cancer screening and treatment programmes, thus supporting evidence-based public health planning [5].

However, there is a lack of literature on survival analysis and prognostic factors of esophageal carcinoma (EC) from India. To the best of our knowledge, this is among the first population-based studies to evaluate EC survival in central India. The primary objective of this study was to estimate the incidence, mortality, and overall survival (OS) of patients with EC registered in the PBCR of central India between 2010 and 2019. The primary endpoint was OS. Secondary objectives were to evaluate the association of age, sex, tumor site, histological subtype, treatment modality, and metastatic status with OS using Kaplan-Meier and Cox proportional hazards analyses.

## Materials and methods

Study cases and cancer registry

In 2002, as part of the National Cancer Registry Programme (NCRP), a PBCR was established in the study district of central India. This study utilized data from the PBCR of a district in central India. The registry functions under the NCRP of the Indian Council of Medical Research (ICMR). Since 2010, the Department of Pathology at a regional tertiary care hospital has coordinated registry activities in the study district. The PBCR systematically records all newly diagnosed cancer cases occurring among residents of both urban and rural areas who must have lived in one district for at least one year within the registry [5,6].

Data collection

Incidence Data

The PBCR database was used to extract incident cancer cases that were diagnosed between 2010 and 2019. There were a total of 28 reporting sources that contributed to the data set. These included public and private healthcare facilities, universities offering medical degrees, cancer centers, pathology labs, and nursing homes. The regional tertiary care hospital reported about half of the cases that were recorded, with the other half coming from other institutions in the registry area and nearby districts. Reviewing records from various departments, such as pathology, radiology, radiation therapy, inpatient care, and outpatient services, allowed for the collection of data. The data were entered into the PBCR data management software (PBCR DM version 2.1.1) using the standardized PBCR core proforma for demographic, clinical, and pathological information [5,6].

Mortality Data

Mortality data were collected through routine follow-up and through official death records maintained by the registry. Cases of EC were identified according to the International Classification of Diseases for Oncology, Third Edition (ICD-O-3) based on the topographic code C15. Survival status was determined, and OS was estimated through the linkage of mortality records to incident cases [5-7].

Study Design

This is a retrospective, population-based observational cohort study based on data obtained from the PBCR in central India.

Inclusion and Exclusion Criteria

The following are the inclusion criteria: all patients diagnosed with primary EC during the study period (2010-2019) and registered in the PBCR. The following are the exclusion criteria: duplicate records and patients for whom essential survival dates were unavailable. Patients with a documented date of diagnosis and a last known follow-up date were retained in the survival analysis. Patients who were alive at the end of follow-up or had no subsequent documented event were censored at the date of their last confirmed contact. Analyses of clinicopathological variables (tumor site, histological subtype, treatment modality, and metastatic status) were performed using variable-specific complete-case analysis because of missing data for some variables.

Statistical analysis

All statistical analyses were conducted using Statistical Product and Service Solutions (SPSS, version 28; IBM SPSS Statistics for Windows, Armonk, NY). Baseline demographic and clinicopathological characteristics were summarized using appropriate descriptive statistical methods. All 545 incident EC cases and 553 EC deaths registered in the PBCR during 2010-2019 were included in the calculation of incidence and mortality rates, respectively. The mortality count included all EC deaths occurring among residents of the PBCR catchment area during 2010-2019, including deaths among patients diagnosed before 2010 who died during the study period after mortality-incidence linkage according to NCRP/ICMR PBCR procedures.

Crude incidence rate (CR) and crude mortality rate (CMR) were estimated as the number of newly diagnosed cases and deaths, respectively, divided by the corresponding mid-year population, and presented per 100,000 population. Confidence intervals (95%) for these rates were derived assuming a Poisson distribution.

Age-specific incidence (ASpR) and mortality (ASpMR) rates were calculated separately for predefined age categories. To facilitate comparison across populations with different age structures, age-standardized incidence rates (ASIR) and age-standardized mortality rates (ASMR) were estimated using the direct standardization approach. The observed age-specific rates were weighted according to the Segi-Doll World Standard Population, as recommended by the International Agency for Research on Cancer (IARC), and expressed per 100,000 population. The world standard population is defined to reflect the average age structure of the world’s population over the next generation from the year 2000-2025 [6].

ASIR and ASMR were calculated using the direct method of age standardization. Initially, ASpR and ASpMR rates were obtained for each age category by dividing the number of incident cases or deaths by the corresponding age-specific population and expressing the result per 100,000 population. These age-specific rates were subsequently weighted using the Segi-Doll World Standard Population, the reference population recommended by the IARC for international comparisons. The world standard population is defined to reflect the average age structure of the world's population over the next generation from the year 2000 to 2025. The weighted age-specific rates were summed to derive the overall ASIR and ASMR, which were reported per 100,000 population. The age-standardized rate was computed as follows:

$ASR = \Sigma(rᵢ &times; wᵢ) / \Sigmawᵢ$

Here, rᵢ denotes the observed ASpR or ASpMR rate in the ith age group, and wᵢ represents the corresponding standard population weight assigned to that age group. The denominator, Σwᵢ, corresponds to the total standard population weight (100,000) [6].

OS was calculated for 476 eligible patients and defined as the time from the date of diagnosis until death from any cause or the last available follow-up (31 March 2024), whichever occurred first. Survival estimates were generated using the Kaplan-Meier method, while differences between survival curves were assessed with the log-rank (Mantel-Cox) test. Factors independently associated with OS were examined using a multivariable Cox proportional hazards model [6]. OS was analysed using Cox proportional-hazards regression. Univariable analyses were initially performed for the clinicopathological characteristics and treatment-related variables. Variables considered clinically relevant and/or identified a priori as potential prognostic factors were included in the primary multivariable Cox regression model. The primary model was adjusted for age group, sex, tumour site, histological type, and treatment modality. Hazard ratios (HRs) with 95% confidence intervals (CIs) and corresponding p-values were reported. The proportional-hazards assumption was assessed using Schoenfeld residual-based tests. Because a variable-specific violation was identified for the 41-60-year age group (p = 0.020), an age-stratified Cox model was fitted as a sensitivity analysis. In this model, age group was used as the stratification variable, allowing the baseline hazard to differ across age groups, while the effects of the remaining covariates were estimated. The sensitivity model additionally adjusted for metastatic status. Because metastatic status is an important prognostic factor and was considered a potential confounder of the association between treatment and survival, a separate sensitivity analysis adjusted for metastatic status. Four patients with unknown metastatic status were excluded from metastasis-adjusted analyses. To address sparse data, the Distant and Too advanced metastatic categories were combined into an Advanced disease category. Patients with unknown metastatic status were excluded from this sensitivity analysis. Similarly, the 13 patients with unknown treatment status were excluded from treatment-specific Cox regression models rather than being treated as a separate treatment category. These patients remained part of the OS cohort where treatment status was not required for the analysis. The results of the metastasis-adjusted and age-stratified sensitivity analyses were compared with those of the primary model to assess the robustness of the observed associations. All statistical tests were two-sided, and p < 0.05 was considered statistically significant.

Metastatic status was classified using the Population-Based Cancer Registry's Extent of Disease (EOD) classification. Cases were categorized according to the extent of disease documented in the registry as localized disease (confined to the esophagus), regional disease (extension to adjacent tissues and/or involvement of regional lymph nodes), distant metastatic disease (spread to distant organs or non-regional lymph nodes), and advanced disease where extensive disease was documented in the available registry records. Detailed TNM staging information was not consistently available in the registry and therefore could not be included in the analysis.

This study was approved by the Institutional Ethics Committee, Mahatma Gandhi Institute of Medical Sciences (MGIMS), Sevagram, Wardha, India (Approval No. IEC/MS/184/2009).

## Results

A total of 545 patients with primary EC registered in the PBCR between 2010 and 2019 were initially identified. Most cases in the study district were from rural areas, with 349 cases, accounting for 64.03%. Urban areas accounted for 196 patients, representing 35.97% of the total patient population. PBCR reported 9,869 malignancy cases in the specified period, with EC cases accounting for 5.52% of the total cancers reported. A total of 69 cases with no date of diagnosis were excluded during data cleaning. Due to this, 476 cases were considered, comprising 244 males (51.26%) and 232 females (48.73%) (Figure 1). The male-to-female ratio was 1.05:1 (Table 1). For survival analysis, 476 patients with complete survival-related information were included.

**Figure 1:**
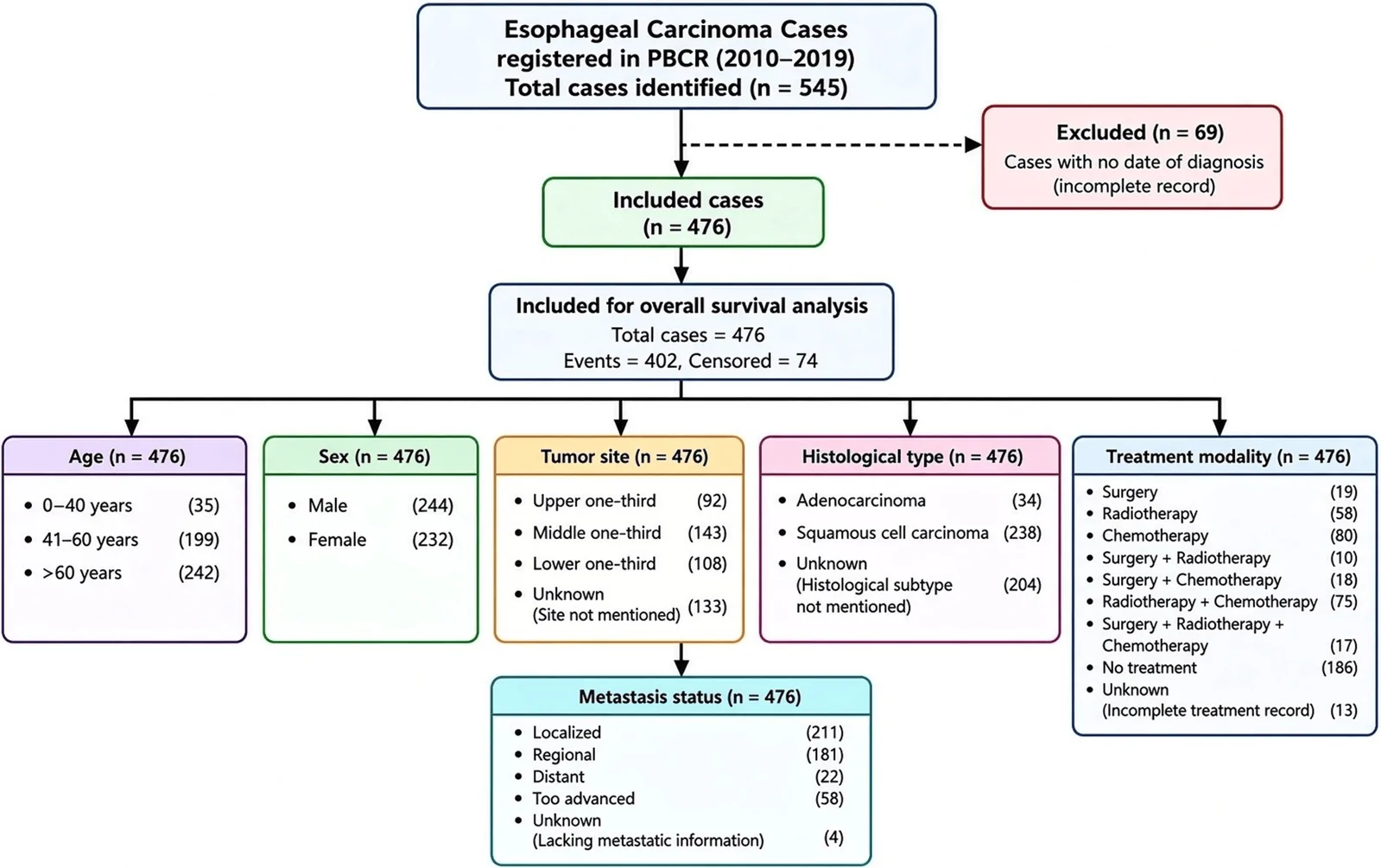
Flow diagram showing esophageal cancer cases included and excluded in the study for various parameter and analysis Variable-specific complete-case analyses were performed for tumor site (n = 343), histology (n = 272), treatment (n = 463), and metastatic status (n = 472).

**Table 1:** Kaplan-Meier estimates of median overall survival according to clinicopathological characteristics in patients with esophageal cancer (n = 476) EC = Esophageal cancer; CI: Confidence interval;  S = Surgery; R = Radiotherapy; C = Chemotherapy Median overall survival was estimated using the Kaplan-Meier method. Sample sizes vary across variables because analyses were restricted to patients with complete data available for the respective variable.

Variables	Total number of cases (n)	Number of events	Number censored	Median survival (95% CI), days
Overall survival	476	402	74	178.33 (115.49-234.48)
Age				
0-40 years	35	30	5	185.12 (106.18-263.81)
41-60 years	199	161	38	193.22 (166.89-231.32)
>60 years	242	211	31	157.42 (125.28-185.73)
Sex				
Male	244	208	36	168.00 (123.25-212.74)
Female	232	194	38	169.00 (136.66-201.34)
Tumor site				
Upper one-third	92	83	9	153.00 (111.68-206.27)
Middle one-third	143	113	30	193.00 (135.97-250.03)
Lower one-third	108	93	15	169.00 (99.73-226.27)
Unknown (site is not mentioned)	133	NA	NA	NA
Histological type				
Adenocarcinoma	34	31	3	126.00 (28.85-223.14)
Squamous cell carcinoma	238	198	40	177.00 (137.32-216.69)
Unknown (histological subtype not mentioned)	204	NA	NA	NA
Treatment modality				
Surgery	19	17	2	71.00 (4.17-137.83)
Radiotherapy	58	56	2	160.00 (115.22-204.78)
Chemotherapy	80	68	12	145.00 (94.60-195.40)
Surgery + radiotherapy	10	9	1	186.00 (136.42-235.69)
Surgery + chemotherapy	18	13	5	289.00 (0.00-706.86)
Radiotherapy + chemotherapy	75	61	14	333.00 (142.53-523.47)
Surgery + radiotherapy + chemotherapy	17	15	2	298.00 (196.23-381.77)
No treatment	186	156	30	110.00 (80.60-139.40)
Unknown (incomplete treatment record)	13	NA	NA	NA
Metastasis status				
Localized	211	163	48	232.00 (159.59-304.41)
Regional	181	157	24	199.00 (166.38-231.61)
Distant	22	20	2	68.00 (54.21-81.79)
Too advanced	58	58	0	28.00 (15.56-40.44)
Unknown (lacking metastatic information)	04	NA	NA	NA

The availability of clinicopathological variables varied because PBCR data were compiled from multiple reporting sources. Consequently, complete information was not available for all registered cases. Age and sex information were available for all 476 patients included in the survival analysis. Tumor site was available for 343 patients, while 133 cases had unspecified anatomical subsite information. Histological subtype was available for 272 patients, whereas 204 cases lacked definite histopathological subtype information. Treatment modality was documented for 463 patients, while treatment information was unavailable for 13 patients due to incomplete treatment records or missing documentation from reporting institutions. A total of 186 cases did not receive any specific treatment. Metastatic status was available for 472 patients, with four cases having unknown metastatic status. Thus, for variables, the number of cases differed: 343 for tumour site, 272 for histological subtypes, 463 for treatment, and 472 for metastatic status (Tables 1-2). To maximise the use of available information, analyses involving clinicopathological variables were performed using complete-case (available-case) analysis, whereby only patients with complete information for the variable under investigation were included. Histopathological classification was available for 272 patients. Of these, 238 (87.5%) had squamous cell carcinoma (SCC), whereas 34 (12.5%) had AC (Table 1). Histological subtype did not demonstrate a significant relationship with OS in either the univariable or multivariable Cox regression models. Using AC as the reference category, patients with SCC had a lower estimated risk of mortality on univariable analysis (HR: 0.81, 95% CI: 0.55-1.17); however, this difference was not statistically significant (p = 0.255). After adjustment for other clinicopathological variables, SCC continued to show no independent association with OS (adjusted HR: 0.84, 95% CI: 0.57-1.25; p = 0.400) included in that specific analysis. Consequently, the denominator varied across different analyses according to data availability (Table 2).

**Table 2:** Evaluation of clinicopathological data completeness and unknown values in the primary survival cohort

Variables	Available n (%)	Unknown n (%)	Total
Tumour site	343 (72.1)	133 (27.9)	476
Histology	272 (57.1)	204 (42.9)	476
Treatment	463 (97.3)	13 (2.7)	476
Metastatic status	472 (99.2)	4 (0.8)	476

The overall CR and CMR for EC were 4.22 (95% CI: 3.73-4.71 per 100,000) for males and 3.99 (95% CI: 3.50-4.48 per 100,000) for females, with 3.59 (95% CI: 3.24-3.93 per 100,000) for males and 2.9 (95% CI: 2.55-3.24 per 100,000) for females per 100,000 population, respectively (Figure 2). The ASIR of overall EC cases was 3.99 per 100,000 population (95% CI: 3.50-4.47), while the ASMR was 3.29 per 100,000 population (95% CI: 2.94-3.64). The maximum ASIR was found in the 65-69-year age group (25.79), followed by the 70-74-year age group (25.03) for males (Figure 3).

**Figure 2:**
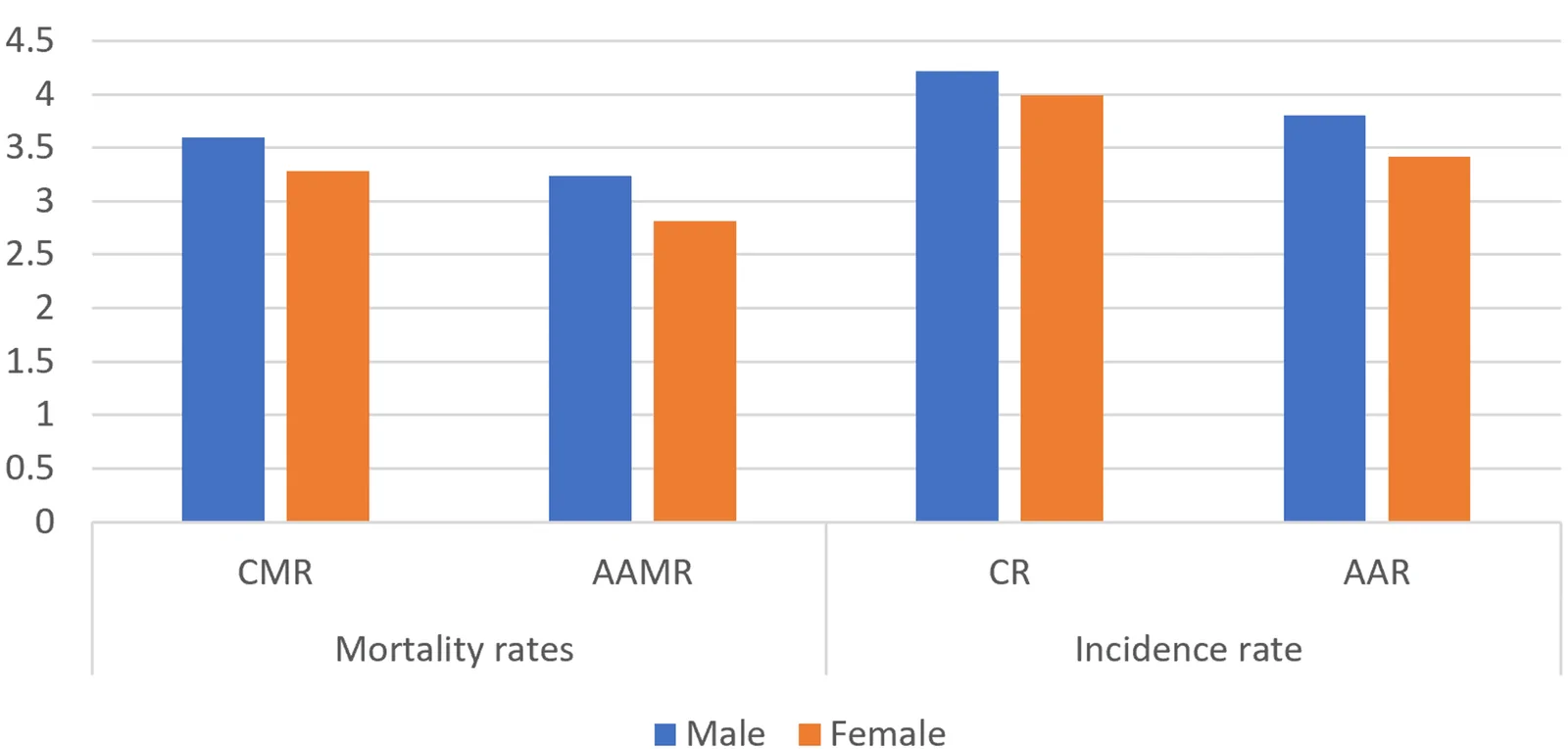
Crude incidence rate (CR), age-standardized incidence rate (AAR), crude mortality rate (CMR), and age-standardized mortality rate (AAMR) of esophageal cancer at our population-based cancer registry

**Figure 3:**
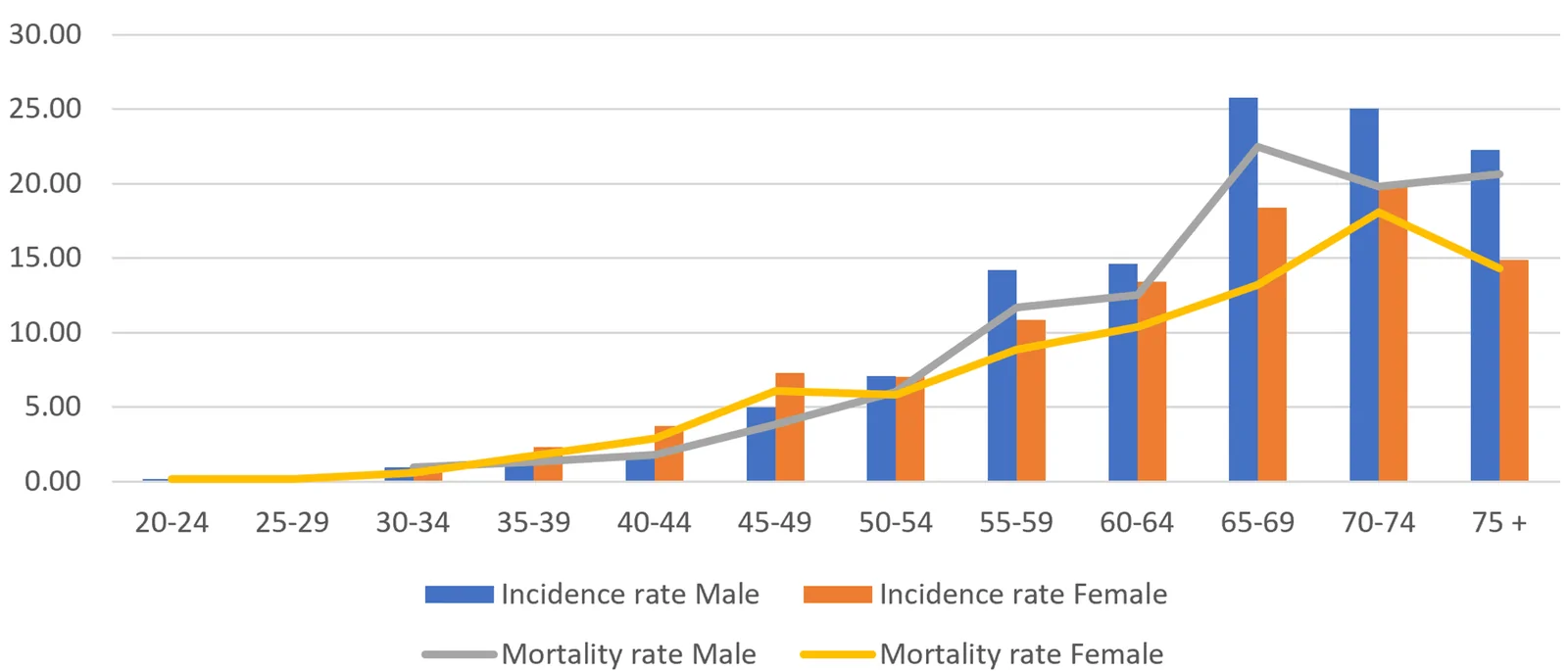
Age and gender-wise incidence and mortality rates of cases

Cox proportional hazards regression was used to investigate the relationship between clinicopathological variables and OS in patients with EC (Figure 4). The primary multivariable Cox proportional hazards model included 463 patients with complete information on treatment modality and adjusted for age group, sex, tumor site, and histological subtype. Univariable analysis did not identify age, sex, or histological subtype as significant predictors of survival.

**Figure 4:**
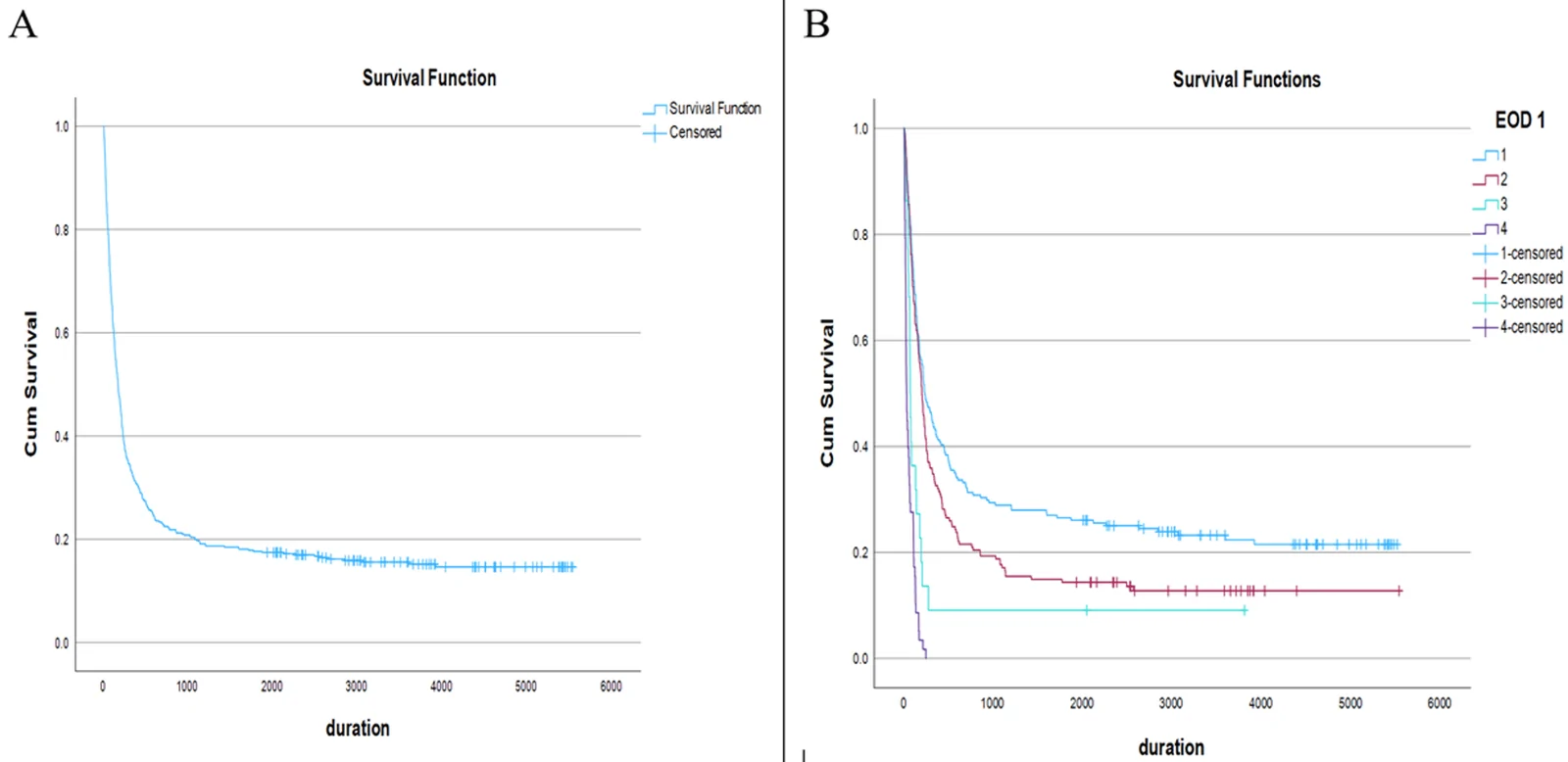
(A) Showing the overall survival among esophageal cancer (EC) patients. (B) Survival graph amongst the EC patients depending on the spread of tumor (EOD, i.e., metastatic status of patient) 1 = Localized, 2 = Regional, 3 = Distant, 4 = Too advanced

Among the 476 patients included in the survival analysis, the largest proportion belonged to the older than 60 years age group (242 cases, Figure 5), followed by those aged 41-60 years (199 cases) (Table 1). Using patients aged 0-40 years as the reference category, no significant difference in the risk of death was observed for patients aged 41-60 years (HR: 0.95, 95% CI: 0.65-1.41; p = 0.815) or older than 60 years (HR: 1.10, 95% CI: 0.75-1.61; p = 0.641). Similarly, mortality risk did not differ significantly between male and female patients (HR: 0.96, 95% CI: 0.79-1.16; p = 0.645) (Table 3).

**Figure 5:**
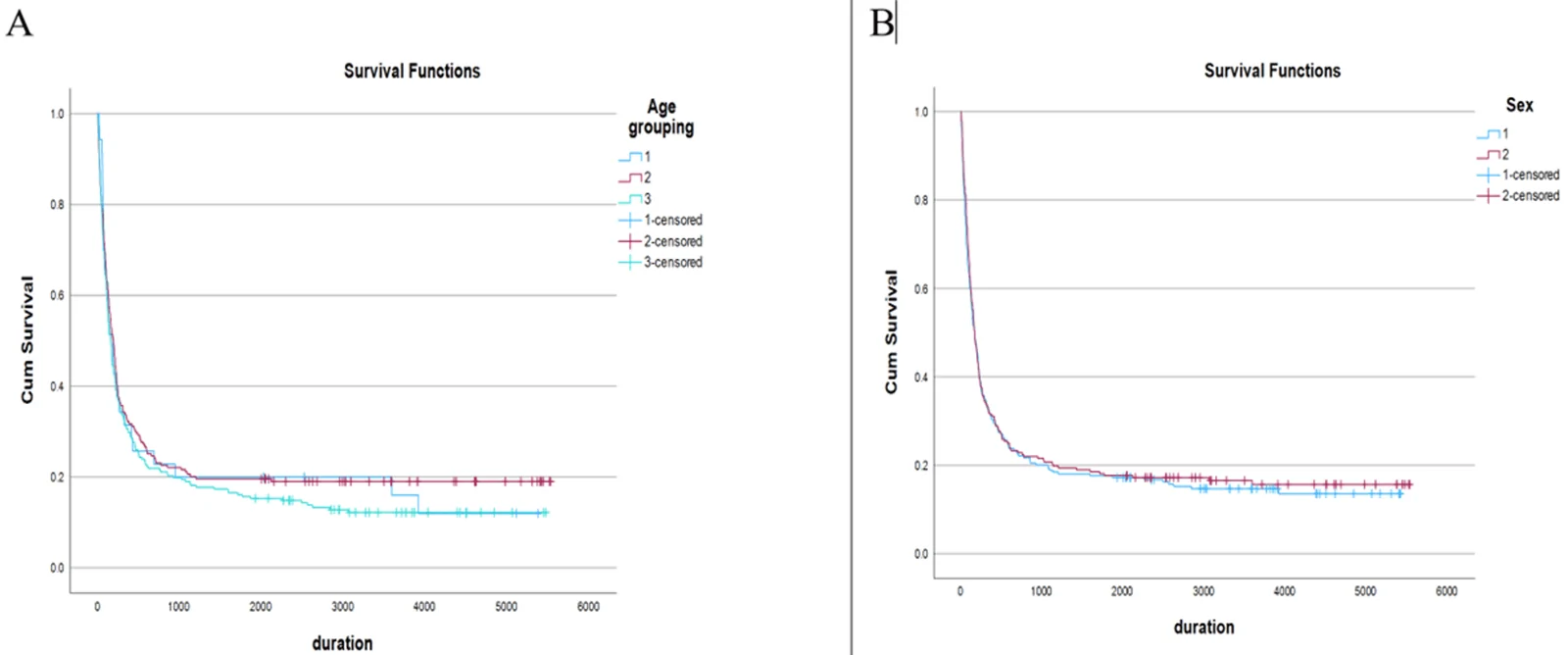
(A) Survival graph amongst the EC patients depending on age group (1 = <40 years, 2 = 41-60 years, 3=>60years). (B) Survival graph amongst the gender of the EC cases (1 = male, 2 = female)

**Table 3:** Univariable and multivariable Cox proportional hazard regression analysis of overall survival Note: The primary multivariable Cox regression model adjusted for age, sex, tumor site, histological type, and treatment modality. To account for disease extent, a sensitivity model additionally adjusted for metastatic status, collapsing "Distant" and "Too advanced" categories into a single "Advanced disease" group to handle sparse data and complete separation. To address a proportional-hazards assumption violation in the 41-60 age group (Schoenfeld residual test, $p = 0.020$), an age-stratified model was also fitted by stratifying on age group and adjusting for metastatic status, which precludes estimating hazard ratios for age itself. Exclusions were applied based on data availability: 13 patients with unknown treatment status were omitted from treatment-specific Cox models while retained in the main overall survival cohort, and four patients lacking metastatic status were excluded exclusively from metastasis-adjusted analyses. All exponentiated coefficients represent hazard ratios (HR) with accompanying 95% confidence intervals (CI) and p-values.

Parameters	Univariable			Primary multivariable			Metastasis-adjusted multivariable (sensitivity analysis)			Age-stratified + metastasis-adjusted multivariable (sensitivity analysis)		
Characteristics	HR	95% CI	p	HR	95% CI	p	HR	95% CI	p	HR	95% CI	p
Age												
0-40 years	1.00			1.00			1.00			1.00		
41-60 years	0.95	(0.64, 1.41)	0.815	0.89	(0.60,1.33)	0.581	0.81	(0.54, 1.21)	0.308			
>60 years	1.01	(0.74, 1.61)	0.641	1.01	(0.68,1.50)	0.949	0.96	(0.65, 1.43)	0.857			
Sex												
Male	1.00			1.00			1.00			1.00		
Female	0.96	(0.79, 1.16)	0.645	0.88	(0.72,1.08)	0.210	0.93	(0.76, 1.15)	0.518	0.94	(0.76, 1.15)	0.530
Tumor Site												
Upper one-third	1.00			1.00			1.00			1.00		
Middle one-third	0.74	(0.56, 0.98)	0.036	0.81	(0.61,1.09)	0.161	0.74	(0.56, 1.00)	0.048	0.74	(0.55, 1.00)	0.049
Lower one-third	0.94	(0.70, 1.26)	0.673	1.05	(0.77,1.44)	0.755	0.99	(0.72, 1.37)	0.974	0.99	(0.72, 1.37)	0.985
Unknown	0.89	(0.68, 1.19)	0.455	0.93	(0.68,1.26)	0.618	0.77	(0.56, 1.06)	0.105	0.78	(0.57, 1.08)	0.132
Histological Type												
Adenocarcinoma	1.00			1.00			1.00			1.00		
Squamous cell carcinoma	0.81	(0.55, 1.17)	0.255	0.84	(0.57,1.25)	0.400	0.85	(0.57, 1.26)	0.422	0.86	(0.58, 1.27)	0.446
Unknown	0.84	(0.57, 1.23)	0.364	0.84	(0.56,1.26)	0.403	0.85	(0.57, 1.28)	0.437	0.87	(0.58, 1.30)	0.485
Treatment Modality												
Surgery	1.00			1.00			1.00			1.00		
Radiotherapy	0.95	(0.55, 1.64)	0.860	0.90	(0.52,1.57)	0.714	0.75	(0.43, 1.30)	0.303	0.74	(0.42, 1.29)	0.286
Chemotherapy	0.75	(0.44, 1.28)	0.297	0.71	(0.41,1.23)	0.220	0.60	(0.35, 1.03)	0.066	0.59	(0.34, 1.02)	0.059
Surgery + Radiotherapy	0.83	(0.37, 1.86)	0.644	0.67	(0.29,1.55)	0.354	0.61	(0.26, 1.43)	0.256	0.59	(0.25, 1.37)	0.221
Surgery + Chemotherapy	0.51	(0.25, 1.05)	0.067	0.45	(0.21,0.94)	0.035	0.43	(0.20, 0.90)	0.026	0.42	(0.20, 0.89)	0.023
Radiotherapy + Chemotherapy	0.53	(0.31, 0.91)	0.022	0.48	(0.28,0.85)	0.011	0.38	(0.21, 0.66)	0.001	0.37	(0.21, 0.66)	0.001
Surgery + Radiotherapy + Chemotherapy	0.98	(0.88, 1.16)	0.121	0.51	(0.25,1.05)	0.070	0.49	(0.23, 1.00)	0.051	0.47	(0.23, 0.98)	0.044
No treatment	0.79	(0.48, 1.31)	0.366	0.72	(0.43,1.21)	0.212	0.57	(0.34, 0.96)	0.034	0.56	(0.33, 0.95)	0.03
Unknown	0.28	(0.11, 0.66)	0.004	-	-	-	-	-	-	-	-	-
Metastasis Status												
Localized	1.00						1.00			1.00		
Regional	1.32	(1.06, 1.64)	0.014	-	-	-	1.26	(1.01, 1.59)	0.044	1.26	(1.00, 1.59)	0.046
Advanced	4.47	(3.37, 5.94)	<0.001	-	-	-	4.67	(3.47, 6.27)	0.000	4.57	(3.39, 6.16)	<0.001
Unknown	2.72	(1.01, 7.36)	0.049	-	-	-	-	-	-	-	-	-
Number of patients	476			463			463			463		

The middle third of the esophagus was the most frequently involved tumor location (143 cases), followed by the lower third (108 cases) (Table 1). Patients with tumors in the middle third demonstrated the longest median OS, with a median survival time of 193.22 days (95% CI: 135.97-250.04), compared with tumors arising in the upper or lower third of the esophagus. Tumor location showed a significant association with OS in the univariable Cox model. Relative to tumors in the upper third, tumors located in the middle third were associated with a 26% lower hazard of death (HR: 0.74, 95% CI: 0.56-0.98; p = 0.036). In contrast, tumors in the lower third showed no statistically significant difference in mortality risk (HR: 0.94, 95% CI: 0.70-1.26; p = 0.673). After adjustment for other clinicopathological variables in the multivariable model, the survival advantage associated with middle-third tumors was reduced and was no longer statistically significant (adjusted HR: 0.80, 95% CI: 0.61-1.06; p = 0.127) (Table 3).

Histopathological classification was available for 272 patients. Of these, 238 (87.5%) had SCC, whereas 34 (12.5%) had AC (Table 1). Histological subtype did not demonstrate a significant relationship with OS in either the univariable or multivariable Cox regression models. Using AC as the reference category, patients with SCC had a lower estimated risk of mortality on univariable analysis (HR: 0.81, 95% CI: 0.55-1.17); however, this difference was not statistically significant (p = 0.255). After adjustment for other clinicopathological variables, SCC continued to show no independent association with OS (adjusted HR: 0.84, 95% CI: 0.57-1.25; p = 0.400) (Table 3).

A total of 211 cases showed a tumor localized to the esophagus. Regional metastases were noted in 181 cases. Distant metastases were found in 22 cases. Too advanced cancer was found in 58 cases. The hazard ratio in the spread of EC cases shows marked variation. A clear stepwise increase in mortality risk was observed with advancing disease spread. In univariable analysis, compared with localized disease, regional disease was associated with a modest increase in mortality (HR: 1.32, 95% CI: 1.06-1.64; p = 0.014), while advanced disease was associated with substantially increased mortality (HR: 4.47, 95% CI: 3.37-5.94; p < 0.001) (Table 3).

Among the 463 patients with available treatment information, 277 received one or more treatments, while 186 received no specific treatment. Among treated patients, chemotherapy alone was the most common modality (80 cases), followed by radiotherapy plus chemotherapy (75 cases), radiotherapy alone (58 cases), surgery alone (19 cases), surgery plus chemotherapy (18 cases), combined surgery, radiotherapy, and chemotherapy (17 cases), and surgery plus radiotherapy (10 cases). Among treatment modalities, radiotherapy plus chemotherapy was associated with significantly improved OS in the univariable analysis (HR: 0.53, 95% CI: 0.31-0.91; p = 0.022). After multivariable adjustment, this association remained significant (adjusted HR: 0.48, 95% CI: 0.28-0.85; p = 0.011). Surgery plus chemotherapy also became independently associated with improved survival after adjustment (adjusted HR: 0.45, 95% CI: 0.21-0.94; p = 0.035), whereas radiotherapy alone, chemotherapy alone, surgery plus radiotherapy, surgery plus radiotherapy plus chemotherapy, and no treatment were not significantly associated with survival in the primary multivariable model (Tables 1, 3).

Sensitivity Analyses

In the metastasis-adjusted sensitivity analysis, 463 patients were included. The association between treatment modality and OS remained largely unchanged after adjustment for metastatic status. Compared with the reference treatment group, radiotherapy plus chemotherapy remained significantly associated with better OS (HR: 0.38, 95% CI: 0.21-0.66; p = 0.001). Surgery plus chemotherapy was also associated with better survival (HR: 0.43, 95% CI: 0.20-0.90; p = 0.026). The association for surgery plus radiotherapy plus chemotherapy was borderline statistically significant (HR: 0.49, 95% CI: 0.23-1.00; p = 0.051). Tumors located in the middle one-third of the esophagus also demonstrated significantly lower mortality compared with upper-third tumors (HR: 0.74, 95% CI: 0.56-1.00; p = 0.048).

Metastatic status showed a strong association with OS. Compared with patients with localized disease, those with regional disease had higher mortality (HR: 1.26, 95% CI: 1.01-1.59; p = 0.044), while patients with advanced disease had substantially higher mortality (HR: 4.67, 95% CI: 3.47-6.27; p < 0.001).

The proportional hazards assumption was assessed using Schoenfeld residuals. Although the global test indicated (χ² = 22.61, df = 16, p = 0.125) no evidence of violation of the proportional hazards assumption, the 41-60-year age group showed a statistically significant covariate-specific Schoenfeld residual test (p = 0.020) (Table 4, Figure 6). Therefore, an age-stratified Cox model additionally adjusted for metastatic status was fitted as a sensitivity analysis.

**Table 4:** Tests of the proportional hazards assumption using Schoenfeld residuals Note: ρ (rho), Schoenfeld residual correlation coefficient.

Variable	Category	Schoenfeld correlation (ρ)	χ²	df	P value
Age	0-40 years	-	-	1	-
	41-60 years	-0.1175	5.4	1	0.0201
	>60 years	-0.0372	0.53	1	0.468
Sex	Male	-		1	-
	Female	-0.0124	0.07	1	0.7937
Tumor site	Upper one-third	-	-	1	-
	Middle one-third	-0.0577	1.35	1	0.2448
	Lower one-third	-0.0616	1.69	1	0.1934
	Unknown	-0.0190	0.15	1	0.7015
Histological type	Adenocarcinoma	-	-	1	-
	Squamous cell carcinoma	-0.0276	0.27	1	0.601
	Unknown	-0.0081	0.02	1	0.8766
Treatment modality	Surgery	-	-	1	-
	Radiotherapy	0.0586	1.67	1	0.1961
	Chemotherapy	0.0372	0.7	1	0.4021
	Surgery + radiotherapy	0.0363	0.6	1	0.439
	Surgery + chemotherapy	0.013	0.09	1	0.7668
	Radiotherapy + chemotherapy	0.0741	2.79	1	0.0946
	Surgery + radiotherapy + chemotherapy	0.0765	2.56	1	0.1097
	No treatment	0.0117	0.07	1	0.7867
	Unknown	0.0643	1.78	1	0.1818
Global test	-	-	22.61	16	0.1245

**Figure 6:**
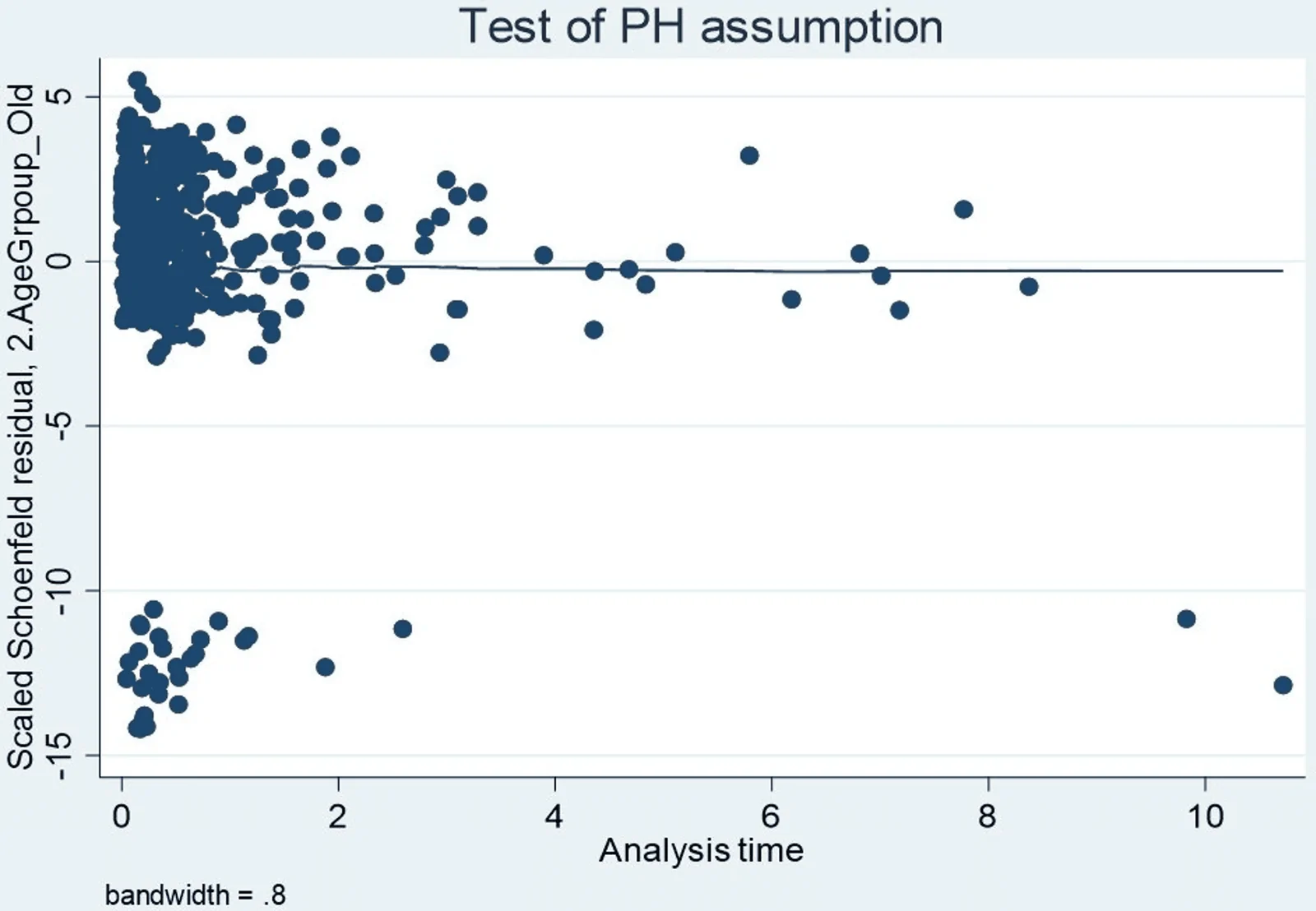
Scaled Schoenfeld residuals for age group two over analysis time EC: Esophageal cancer

The age-stratified model produced results highly consistent with the metastasis-adjusted model. Radiotherapy plus chemotherapy remained significantly associated with improved OS (HR: 0.37, 95% CI: 0.21-0.66; p = 0.001). Surgery plus chemotherapy also remained significantly associated with better survival (HR: 0.42, 95% CI: 0.20-0.89; p = 0.023), while surgery plus radiotherapy plus chemotherapy became statistically significant (HR: 0.47, 95% CI: 0.23-0.98; p = 0.044). Middle-third tumors continued to demonstrate lower mortality than upper-third tumors (HR: 0.74, 95% CI: 0.55-1.00; p = 0.049). Advanced disease remained strongly associated with mortality (HR: 4.57, 95% CI: 3.39-6.16; p < 0.001). Overall, the similarity of the treatment and metastatic status estimates across the primary, metastasis-adjusted, and age-stratified models supports the robustness of the principal findings (Tables 3-4).

The Kaplan-Meier survival analysis demonstrated a progressive decline in OS over time. The estimated probability of survival was 31.3% at one year, 19.7% at three years, 17.4% at five years, and 14.6% at 10 years. Correspondingly, the cumulative probability of death reached 80.3% by three years and 82.6% by five years (Table 5, Figure 4A).

Differences in OS between patient groups were evaluated using the Kaplan-Meier method, with comparisons performed using the log-rank (Mantel-Cox) test. Survival distributions differed significantly according to treatment modality (χ² = 15.421, df = 7, p = 0.031), indicating variation in OS among the different treatment groups (Table 5, Figure 7). Metastatic status also had a significant influence on survival. Patients with different extents of disease spread showed significantly different Kaplan-Meier survival curves (χ² = 162.641, df = 3, p < 0.001) (Table 5, Figure 4B). A significant difference in survival was likewise observed across tumor site categories (χ² = 5.242, p = 0.043) (Table 5, Figure 8A).

**Figure 7:**
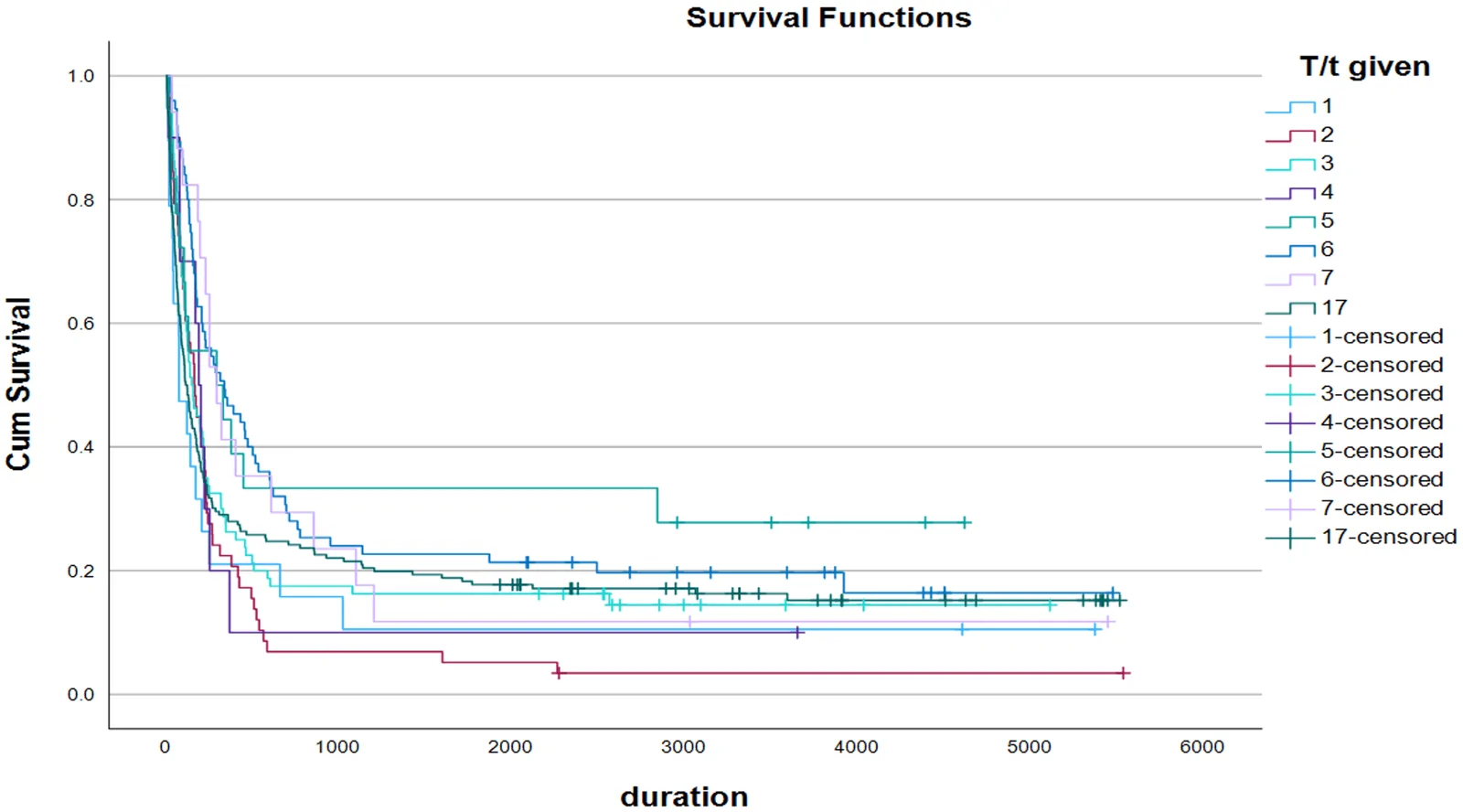
Survival graph among the EC patients depending on the treatment 1 = Surgery (S), 2 = Chemotherapy (C), 3 = Surgery + Radiotherapy (R), 4 = Surgery + Chemotherapy, 5 = Radiotherapy + Chemotherapy, 6 = Chemotherapy + Radiotherapy, 7 = Surgery+ Chemotherapy + Radiotherapy, 17 = No treatment

**Figure 8:**
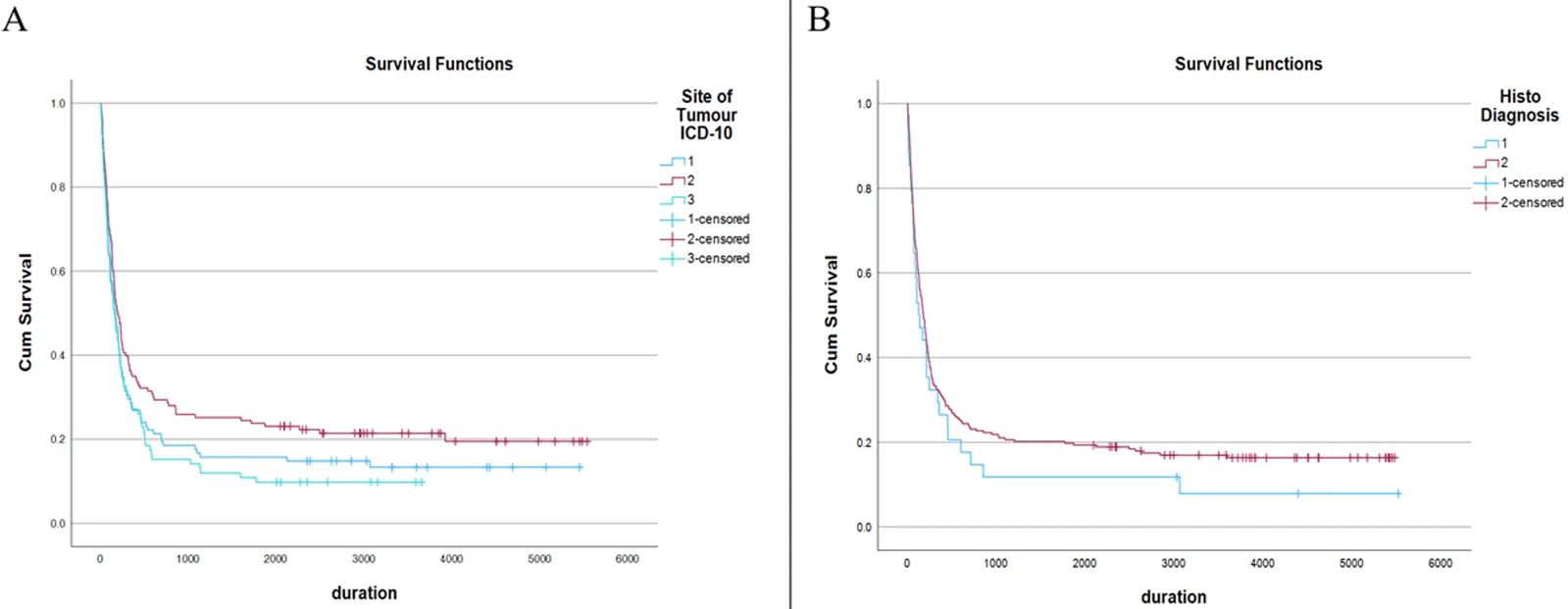
(A) Survival graph amongst the EC patients depending on the site of the tumor (1 = upper 1/3rd of esophagus, 2 = middle 1/3rd of esophagus, 3 = lower 1/3rd of esophagus). (B) Survival graph among the EC patients depending on the histological type of the tumor (1 = adenocarcinoma, 2 = squamous cell carcinoma)

In contrast, Kaplan-Meier survival curves did not differ significantly according to histological subtype (χ² = 1.243, p = 0.265), age group (χ² = 1.738, p = 0.419), or sex (χ² = 0.221, p = 0.638) (Table 3, Figures 5A, 5B, and 6B). Overall, the Kaplan-Meier analysis identified significant differences in OS according to tumor site, treatment modality, and metastatic status, whereas no significant survival differences were observed for age, sex, or histological subtype. The findings from the Cox proportional hazards regression analysis were consistent with the results of the Kaplan-Meier and log-rank analyses (Tables 3, 5).

**Table 5:** Comparison of the overall survival according to clinicopathological characteristics of patients with esophageal cancer using the log-rank (Mantel-Cox) test EC: Esophageal cancer, NS: Nonsignificant, DF: Degree of freedom, S: Significant, LR: Log rank

Sr. No	Variable	Chi-square value (χ²)	DF	P value
1	Survival outcome depending on age	1.738	2	0.419
2	Survival outcome depending on gender	0.221	1	0.638
3	Survival outcome depending on the tumour site of the EC	5.242	2	0.043
4	Survival outcome depending on the histological type of EC	1.243	1	0.265
5	Survival outcome depending on the treatment given	15.421	7	0.031
6	Survival outcome depending on the metastasis status	162.641	3	<0.001

## Discussion

Substantial geographic differences in the burden of EC are evident across the world. GLOBOCAN 2022 data show that the lowest incidence rates are reported from Western Africa and Central America, whereas Eastern Asia and parts of Southern and Eastern Africa experience the greatest disease burden. More than half of all newly diagnosed EC cases occur in Eastern Asia, with China contributing the majority of cases within this region. In the present study, we found an age-standardized incidence rate of EC of 3.8 per 100,000 for males and 3.42 per 100,000 for females, which is lower than the worldwide statistics (6.3 per 100,000) and Indian statistics (4.7 per 100,000) [8] (Figures 2-3).

PBCR data from India (2012-2016) also demonstrate considerable regional variation in the occurrence of EC. The East Khasi Hills district of Meghalaya reported the highest age-adjusted incidence rates in both sexes, followed by Aizawl in Mizoram, highlighting the concentration of disease in northeastern India. The data from the ICMR explain the marked variation in the incidence of EC. The data show that cancers, especially ECs linked to tobacco, are very common across different regions of India. The highest rates are reported from the northeastern states, particularly Meghalaya and Assam. The smoking habits of people in Northeast India are distinct from those in the rest of the country [9]. In the present study, there were 349 cases (64.04%) in the rural area and 196 cases (35.96%) in the urban population. Because of poor socioeconomic conditions, low health awareness, and limited access to healthcare services, the incidence of EC is higher in rural areas, which explains why EC is more common there [10]. Study data analysis revealed that the 60-69 age group had the highest number of EC cases (171), followed by the 70-79 age group with 119 cases, and the 50-59 age group with 117 cases (Figure 3), which is comparable to research conducted in Kashmir, India [11], while, in contrast, Choksi et al. [9] reported that patients from Mumbai had presented at a younger age (Figure 5A). Study data analysis revealed that the 60-69-year age group had the highest number of EC cases, followed by the 70-79-year and 50-59-year age groups, which is comparable to findings from Kashmir, India.

GLOBOCAN 2022 [1] estimates indicate that approximately 70% of EC cases occur in men [1]. The higher incidence among males has commonly been attributed to the greater prevalence of established risk factors, particularly tobacco consumption and alcohol use, in many populations [12].

However, the current study's findings contradict this, showing that the male-to-female ratio is 1.05:1, indicating a relatively balanced gender distribution (Table 1, Figure 5B). This is consistent with the gender distribution of cases observed in all EC patients in Africa (1.5:1) and in other high-risk groups in developing countries such as China and Iran [13-14]. This M: F ratio is much lower than that found in industrialized countries [15], probably due to exposure to risk factors such as dietary habits and other unknown factors, similar to those observed in males and females in our study [15].

Histopathological analysis in the present study showed that SCC was the predominant subtype (Table 1, Figure 8B). This observation is consistent with the epidemiological pattern reported in East Asia and other regions with a high incidence of EC, where SCC remains the most common histological type. Conversely, esophageal AC is more frequently encountered in many Western countries, and its incidence has increased over recent decades [16-19]. The etiological factors associated with the two major histological subtypes differ substantially. SCC is strongly associated with tobacco use and alcohol consumption, whereas AC is more closely related to obesity, gastroesophageal reflux disease, and Barrett's esophagus [16]. In the current study, SCC patients had greater median survival than AC patients (Table 1, Figure 8B). Histological type was not significantly associated with OS (p = 0.265). Although patients with SCC had a numerically lower hazard of death than those with AC, the difference did not reach statistical significance. This finding is consistent with the nonsignificant log-rank comparison between the two histological groups. The relatively small number of patients with AC in the available histological dataset should be considered when interpreting this result.

In the present study, a large proportion of patients presented with advanced-stage disease. Overall, 55.29% of patients had disease extending beyond localized disease at diagnosis, including patients with regional spread, distant metastasis, and locally advanced disease. Similar findings have been reported by Baidya et al. [10] and McHembe et al. [20], indicating that late presentation remains a major challenge in EC. The prognostic importance of disease extent observed in the present study is consistent with findings from both the Greater Mumbai and Bhopal PBCRs. The Mumbai registry identified regional spread and distant metastasis as independent predictors of mortality, with the hazard of death increasing progressively with advancing disease extent [21]. Similarly, the Bhopal PBCR demonstrated a marked decline in five-year survival with increasing disease extent [22]. Patients with locoregional disease had significantly better OS than those with metastatic disease (p < 0.001). Median survival declined progressively from localized disease to regional disease, distant metastasis, and too advanced disease, and the Kaplan-Meier and Cox regression analyses consistently demonstrated metastatic status as the strongest predictor of mortality. These findings across three independent Indian PBCRs emphasize that disease extent at diagnosis remains the most important determinant of survival in EC. Comparison with earlier Indian PBCR studies suggests modest improvement in long-term survival over the past two decades; however, direct comparisons should be interpreted cautiously because of differences in study period, registry population, staging practices, and treatment availability. Despite these improvements, survival remains poor because a substantial proportion of patients continue to present with advanced or metastatic disease. These observations underscore the importance of early diagnosis, accurate staging, timely referral, and improved access to multidisciplinary treatment for patients with EC in rural and semi-urban India.

The one-year, three-year, and five-year OS rates in the present study were 31.3%, 19.7%, and 17.4%, respectively (Table 5, Figure 4A). These survival rates were higher than those reported by Baidya et al. [10] but lower than those reported by Bhandari et al. [23], which may reflect the differences in patient characteristics, disease stage at presentation, and treatment practices across the study populations. Comparison with earlier Indian PBCR studies provides important temporal context. The Greater Mumbai PBCR (1987-1991) [21] reported observed survival rates of 32.7%, 13.6%, and 9.7% at one, three, and five years, respectively, whereas the Bhopal PBCR (1991-1995) [22] reported absolute survival rates of 36.2%, 12.9%, and 3.1% at the same time points. Although one-year survival in the present study was comparable with both registries, three-year and five-year survival were substantially higher than those reported from Mumbai [21] and Bhopal [22]. This improvement may reflect advances in diagnostic evaluation, staging procedures, multidisciplinary treatment, supportive care, and cancer registry follow-up over the intervening two decades. Nevertheless, direct comparison should be interpreted cautiously because the three registries differed in study period, patient population, inclusion criteria, and treatment availability (Table 5, Figure 4A).

Only 19 patients (4.10%) underwent surgical resection in the current study. This low figure is due to the advanced stage of presentation. The maximum median survival in days was observed with chemo-radiation (Table 1, Figure 7). Treatment modality was significantly associated with survival on both univariable and multivariable Cox analyses for selected treatment categories. Radiotherapy combined with chemotherapy was consistently associated with better OS across the primary multivariable model and both sensitivity analyses, suggesting that multimodal non-surgical therapy was associated with improved survival in this registry cohort. Surgery combined with chemotherapy also remained significantly associated with improved survival after adjustment, supporting the potential benefit of combined treatment strategies in appropriately selected patients (Tables 3, 5, Figure 7). However, this finding should be interpreted cautiously because treatment allocation in a retrospective cohort is not random. Treatment choice is influenced by disease stage, metastatic status, operability, performance status, comorbidities, treatment availability, and other clinical factors. Consequently, the observed association should not be interpreted as evidence of a causal survival benefit of radiotherapy plus chemotherapy. Findings in the present study are consistent with the Greater Mumbai PBCR, which also identified treatment modality as an important determinant of survival. Patients receiving combined-modality treatment had better survival than several single-treatment groups, although disease extent remained an important independent predictor of outcome [21].

The metastasis-adjusted and age-stratified sensitivity analyses produced hazard ratio estimates that were highly consistent with the primary multivariable model. This consistency strengthens the robustness of the observed associations between treatment modality and OS. The age-stratified analysis was performed because the Schoenfeld residual test suggested a possible covariate-specific departure from the proportional hazards assumption for the 41-60-year age group, despite the absence of a significant global violation. The similarity of the estimates across models indicates that this isolated finding did not materially influence the study conclusions.

Age was not significantly associated with OS in the present cohort on either log-rank analysis or multivariable Cox regression, despite patients aged 41-60 years having the longest median survival and those aged >60 years showing the shortest median survival. The observed survival differences across age groups were not statistically significant, indicating that age was not an independent predictor of mortality in this PBCR cohort. These findings differ from earlier Indian population-based studies from Greater Mumbai [21] and Bhopal [22], which reported progressively poorer survival with advancing age and the worst outcomes among patients aged ≥75 years. The lack of an independent age effect in our study suggests that poorer survival in older patients is more likely related to advanced disease at presentation, higher comorbidity burden, and reduced likelihood of receiving curative multimodality treatment rather than chronological age alone.

Tumor location showed a modest but consistent association with prognosis. Tumors arising in the middle one-third of the esophagus were associated with lower mortality than upper-third tumors in the univariable analysis and remained significant after adjustment for metastatic status and in the age-stratified sensitivity model (Table 3). However, this association was not statistically significant in the primary multivariable model, suggesting that the effect of tumor location may be partially influenced by differences in disease extent or treatment patterns.

Recent advances in molecular imaging have expanded the role of positron emission tomography/computed tomography (PET/CT) beyond conventional staging in EC. The recent review of molecular imaging in gastrointestinal malignancies highlighted that 18F-fluorodeoxyglucose (18F-FDG) PET/CT is increasingly used for initial staging, assessment of nodal and distant metastases, evaluation of response to neoadjuvant therapy, detection of residual or recurrent disease, and post-treatment surveillance in esophageal carcinoma. In addition to FDG-based imaging, emerging tracers such as 68Ga-fibroblast activation protein inhibitor (68Ga-FAPI) have shown promise for improved tumor characterization and prognostic stratification. Quantitative PET parameters, including maximum standardized uptake value (SUVmax), metabolic tumor volume (MTV), and total lesion glycolysis (TLG), have been associated with OS and progression-free survival in patients with EC, while early evidence suggests that 68Ga-FAPI PET/CT may better predict treatment response and disease outcomes in locally advanced disease. Although molecular imaging was not evaluated in the present population-based registry study, these evolving techniques may enhance risk stratification and support more individualized management strategies in future prospective studies of EC in India [24].

The present study has several strengths. It is based on data from a PBCR, providing a representative overview of EC incidence, mortality, and survival in a predominantly rural population of central India. The study included a relatively large cohort of patients diagnosed over 10 years, allowing assessment of long-term survival patterns. Standard epidemiological methods, including Kaplan-Meier survival analysis and Cox proportional hazards regression, were employed to evaluate prognostic factors associated with OS. In addition, prespecified sensitivity analyses, including a metastasis-adjusted multivariable Cox model and an age-stratified Cox model, were performed to assess the robustness of the primary findings and address potential confounding by disease extent and the observed proportional-hazards assumption violation for the 41-60-year age group. The consistency of hazard ratio estimates across the primary and sensitivity models supports the robustness and internal validity of the study findings. The use of data collected from multiple reporting sources within the registry enhances the generalizability of the findings within the registry catchment area. Furthermore, the study addresses an important evidence gap regarding population-based survival outcomes of EC in rural India, where such data remain limited.

This study has several limitations. First, its retrospective design and use of data from a single PBCR may limit the generalizability of the findings. Second, missing information on tumor site, histology, treatment, and metastasis reduced the sample size for some analyses and may have introduced treatment bias. Treatment allocation was not randomised and may have been influenced by disease stage and patient fitness. Registry data were dependent on the completeness and accuracy of reporting institutions. Third, the absence of detailed TNM staging, comorbidities, performance status, and treatment intent limited adjustment for potential confounding, particularly in the analysis of treatment outcomes. Residual confounding, particularly confounding by disease severity and treatment indication, cannot be excluded. The median follow-up duration was not estimated using the reverse Kaplan-Meier method. Despite these limitations, the study provides valuable population-based evidence on EC survival in rural central India.

## Conclusions

This population-based study demonstrates that EC is associated with a high mortality burden and poor OS in central India. Early diagnosis, timely staging, and appropriate management are essential to improve survival outcomes and reduce presentation at advanced stages. Metastatic status was associated with the strongest prognostic factor, with progressively worse survival observed from localized to regional, distant, and too advanced disease. Tumor site was significantly associated with OS in Kaplan-Meier and univariable Cox analyses, but it did not remain an independent predictor after multivariable adjustment and remained significant after adjustment for metastatic status and in the age-stratified sensitivity model. Radiotherapy plus chemotherapy and surgery plus chemotherapy were independently associated with lower mortality after multivariable adjustment. In contrast, age, sex, and histological subtype were not significantly associated with OS. These findings emphasize the need for strengthened cancer awareness, earlier detection, accurate assessment of disease extent, and improved access to multimodal treatment in rural central India.
